# Micronucleus production, activation of DNA damage response and cGAS-STING signaling in syncytia induced by SARS-CoV-2 infection

**DOI:** 10.1186/s13062-021-00305-7

**Published:** 2021-10-21

**Authors:** He Ren, Chaobing Ma, Haoran Peng, Bo Zhang, Lulin Zhou, Yan Su, Xiaoyan Gao, Hongyan Huang

**Affiliations:** 1 Department of Oncology, Beijing Shijitan Hospital of Capital Medical University, 10 TIEYI Road, Beijing, 100038 China; 2 Department of Microbiology, Second Military Medical University, Shanghai, 200433 China; 3 School of Medicine, Nankai University, 94 Weijin Road, Tianjin, 300071 China; 4 CAS Key Laboratory of Mechanical Behavior and Design of Materials, Department of Modern Mechanics, University of Science and Technology of China, Hefei, 230022 China

**Keywords:** SARS-COV-2, COVID-19, Syncytia, Cell fusion, Micronuclei, DNA damage signaling, γH2Ax, cGAS

## Abstract

SARS-CoV-2 infection could cause severe acute respiratory syndrome, largely attributed to dysregulated immune activation and extensive lung tissue damage. However, the underlying mechanisms are not fully understood. Here, we reported that viral infection could induce syncytia formation within cells expressing ACE2 and the SARS-CoV-2 spike protein, leading to the production of micronuclei with an average rate of about 4 per syncytium (> 93%). Remarkably, these micronuclei were manifested with a high level of activation of both DNA damage response and cGAS-STING signaling, as indicated by micronucleus translocation of γH2Ax and cGAS, and upregulation of their respective downstream target genes. Since activation of these signaling pathways were known to be associated with cellular catastrophe and aberrant immune activation, these findings help explain the pathological effects of SARS-CoV-2 infection at cellular and molecular levels, and provide novel potential targets for COVID-19 therapy.

## Introduction

The pandemic of 2019 novel coronavirus disease (COVID-19), caused by the infection of severe acute respiratory syndrome coronavirus-2 (SARS-CoV-2), has posed a severe threat to global public health [1]. As of September 3, 2021, the cumulative number of COVID-19 cases globally surpassed 2.1 billion, and more than 4.5 million people died according to data from World Health Organization. The situation is still not optimistic with the continuous evolution of the virus, leading to the emerging of novel variants that carry on gain of function mutations, such as D614G, N501Y, E484K and K417N, and therefore are more transmissible [1–5].

The binding of the SARS-CoV-2 spike glycoprotein (S) to angiotensin-converting enzyme 2 (ACE2) on host cells was known to mediate membrane fusion and viral host entry, which then initiates a series of immunological and pathological events that promote respiratory distress syndromes [1, 6–8]. SARS-CoV-2 infection could induce syncytia formation of infected cells [9], which was shown to be able to internalize lymphocytes to form cell-in-cell structures [10], a type of unique cellular structure usually prevalent in human tumor tissues [11–13]. The lymphocytes enclosed into the syncytia primarily underwent cell death, contributing to lymphopenia in patients with COVID-19 [14]. The syncytia themselves were recently shown to succumb to cell death by pyroptosis, potentially enhancing inflammation in the infected patients [15]. These results suggested that syncytia might be an important player for the immune dysregulation and pathogenesis of patients with COVID-19.

Thus, we set out to explore molecular events taking place post syncytium formation by utilizing the well-established model of syncytia induced by S-ACE2 interaction. And we found that micronuclei were frequently present in the multinucleate syncytia, and tightly associated with activation of DNA damage signaling and cGAS-STING signaling, which set a plausible basis for aberrant immune activation and extensive tissue damages occurring in severe patients with COVID-19.

## Results

### Presence of micronuclei in syncytia induced by SARS-COV-2 spike expression

**Fig. 1:**
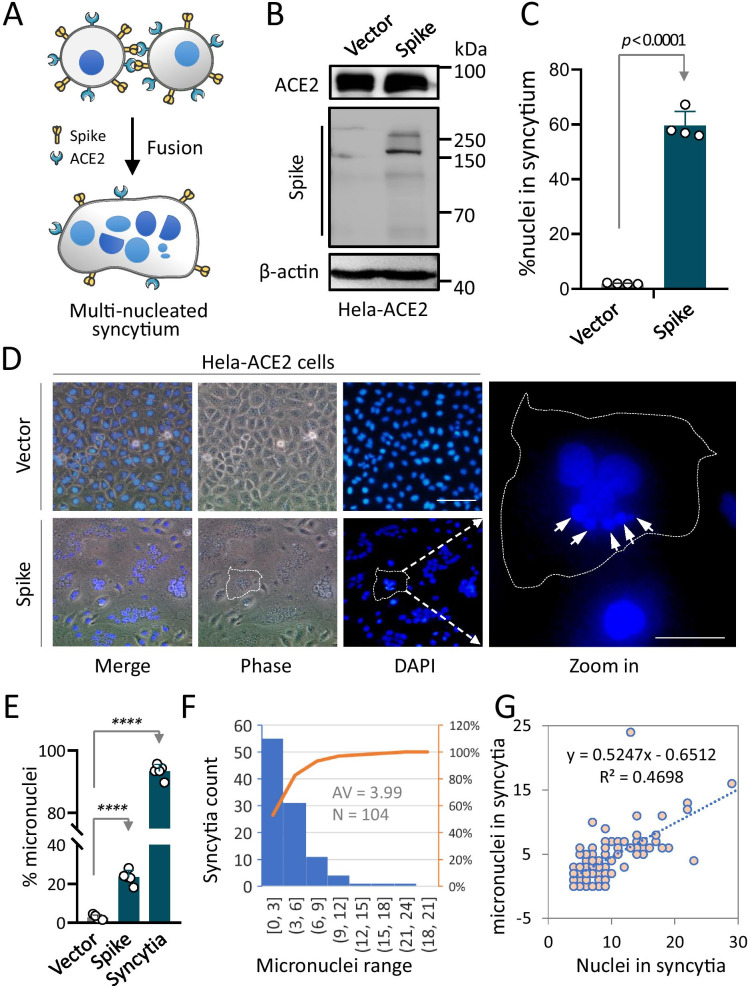
Micronuclei formation in syncytia induced by SARS-CoV-2 spike expression. **a** Schematic representation of cell–cell fusion induced by ACE2-spike interaction to form a multinucleated syncytium. **b** Expression of ACE2 and spike proteins 24 h post transfection detected by Western blot. **c** Quantification of syncytia formation upon expression of spike glycoprotein in Hela-ACE2 cells. Data are mean ± SD of 4 fields (10 × objective lens). ****P < 0.0001. **d** Representative images captured on Hela-ACE2 cells 24 h post transfection. Nuclei were stained with DAPI. White dashed lines depict shape of target syncytium and the white arrows indicate micronuclei. Scale bars: 50 μm for the left images, 20 μm for zoomed images on the right. **e** The formation rate of micronuclei in vector transfection cells, spike protein transfection cells and syncytia, respectively. Data are mean ± SD of 5 fields (20 × objective lens). ****P < 0.0001. **f** Enumeration of micronuclei formation per syncytium. On average, each syncytium cell has 3.99 micronuclei, n = 104. **g** The number of micronuclei were positively associated with the nucleus number of syncytia. Analysis was performed by Spearman rank correlation

To obtain multinucleate syncytia by cell–cell fusion, an event taking place during SARS-CoV-2 infection, we ectopically expressed SARS-CoV-2 S protein in Hela-ACE2 cells as did previously [16], which is expected to promote syncytia formation by enabling mutual interaction between cells expressing both S protein and ACE2 (Fig. 1a). As shown in Fig. 1b–d, expression of S protein (Fig. 1b) effectively induced syncytium formation in Hela-ACE2 cells with a fusion rate of about 60% at 24 h post transfection (Fig. 1c, d), while little syncytia were identified in the vector-transfected cells.

**Fig. 2:**
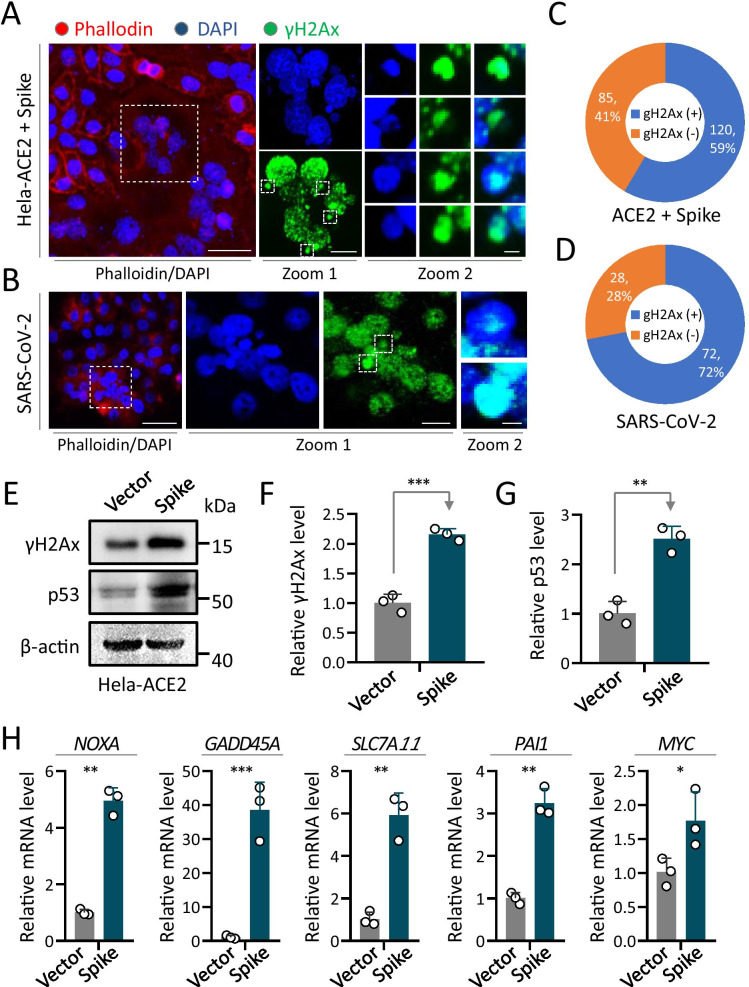
Activation of DNA damage pathway in syncytia induced by SARS-CoV-2 infection and Spike-ACE2 expression. **a** and **b** Immunofluorescent images of Hela-ACE2 cells expressing exogenous SARS-CoV-2 spike glycoprotein (**a**) or infected by SARS-CoV-2 (**b**). Cells were stained with antibodies against γH2Ax protein in green. Zoomed images on the right indicate colocalization between γH2Ax and micronuclei in syncytia. Scale bars: 50 μm for the left image, 20 μm for zoomed images in the middle and 1 μm for multi-channel images on the right. **c** and **d** Quantification of micronuclei γH2Ax foci in Hela-ACE2 cells transfected by spike protein (**c**) or infected by SARS-CoV-2 (**d**). **e**–**g** Expression of γH2AX and p53 proteins in Hela-ACE2 cells 24 h post transfection by Western blot (**e**) and quantification (**f**, **g**). All results were normalized by the expression of β-actin. Data are mean ± SD of triple quantification. **h** Expression of NOXA, GADD45A, SLC7A11, PAI1 and MYC were analyzed by quantitative RT-PCR 24 h post spike transfection in Hela-ACE2 cells

Interestingly, by taking a close look at the syncytia with nuclei stained by the dye of 4,6-diamidino-2-phenylindole (DAPI), we frequently observed some micronuclei that are obviously much smaller in size than the normal nuclei (Fig. 1d, white arrows); the micronuclei were present in a high level in the spike-transfected cells with a frequency of > 23% for all cells, and > 93% for syncytia (Fig. 1e). Within syncytia, there are about 4 micronuclei per syncytium on an average with a range of 0–21, and most of the syncytia contained 0–6 micronuclei (Fig. 1f); and the number of micronuclei appeared to be positively correlated with the number of nuclei (regular size) within each syncytium (Fig. 1g). Moreover, the micronuclei were also observed in Hela-ACE2 cells infected with the authentic SARS-CoV-2 viruses (Fig. 2b), suggesting that the production of micronuclei is a phenomenon associated with syncytia induced by SARS-CoV-2 infection.

### Activation of DNA damage response on the syncytial micronuclei

The production of micronuclei indicates genome instability and potential DNA damages [17], we therefore set to examine the DNA damage status within syncytia. As shown in Fig. 2a, b, most of the syncytial nuclei are positive in γH2Ax (H2Ax with phosphorylation on its Ser139), a marker for DNA damage at the very early phase [18], suggesting that DNA damage occurred within the syncytial nuclei. Intriguingly, the syncytial micronuclei were also positive, seemed to be stronger than the regular nuclei, in γH2Ax with a rate of > 59% positivity in spike-expression cells (Fig. 2c), which was more prominent in syncytia induced by the infection of authentic SARS-CoV-2 virus (Fig. 2b), where more than 72% micronuclei were positive in γH2Ax (Fig. 2d), suggesting that most of the micronuclei contained DNA damages. To investigate whether DNA damage response were activated, we examined the expression of γH2AX and p53 by Western blot, which demonstrated a clear upregulation of these two proteins (Fig. 2e–g). Meanwhile, the expression of p53 target genes downstream of DNA damage response pathway, including NOXA, GADD45A, SLC7A11, PAI1 and MYC [19, 20], were all significantly upregulated along with syncytia formation in Hela-ACE2 cells transfected with S protein, as demonstrated by quantitative RT-PCR (Fig. 2h). Together, these results suggest that the syncytial micronuclei are the sites succumbing to genomic instability and DNA damage.

### Activation of cGAS signaling on the syncytial micronuclei

**Fig. 3:**
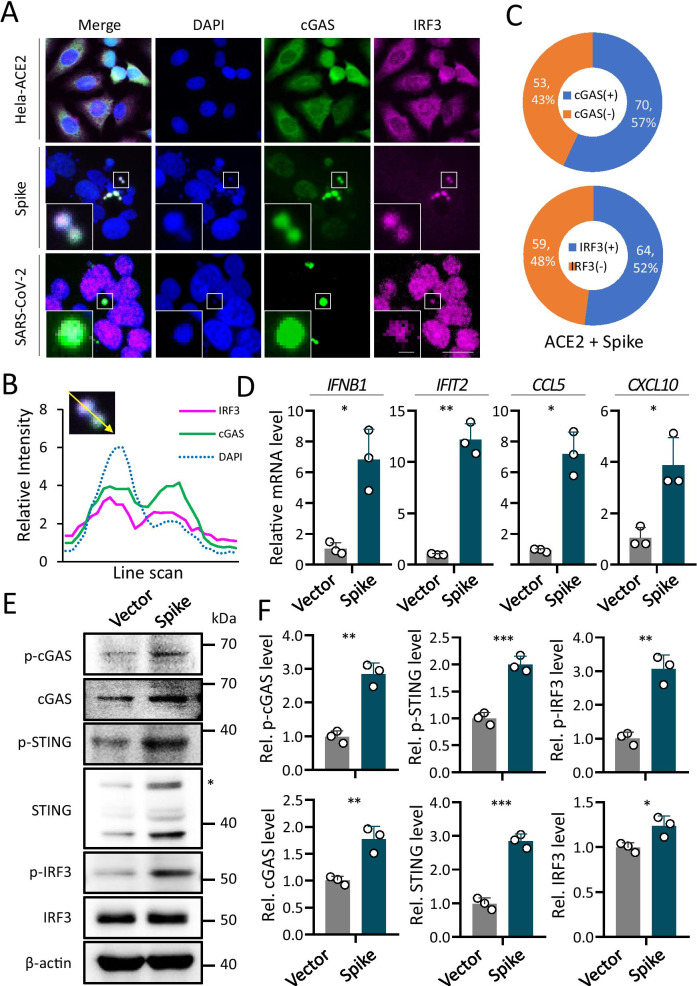
Activation of cGAS pathway in syncytia induced by SARS-CoV-2 infection and Spike-ACE2 expression. **a** and **b** Channeled images (**a**) and line scan analysis (**b**) showed cGAS and IRF3 were co-localized with micronuclei. Scale bars: 1 μm for the inserts in the lower left corner; 20 μm for full images. **c** Quantification of micronuclei positive in cGAS (+) and IRF3 (+) in Hela-ACE2 cells transfected with spike protein. **d** The relative mRNA level of IFIT2, IFNB1, CCL5 and CXCL10, respectively in Hela-ACE2 cells 24 h after transfection. *P < 0.05; **P < 0.01. **e** and **f** The expression of cGAS, STING, IRF3 and their phosphorylated form were increased upon expression of spike protein analyzed by Western blot (**e**) and quantification (**f**). All results were normalized by the expression of β-actin. Data are mean ± SD of triple quantification. *P < 0.05; **P < 0.01; ***P < 0.001

Since cGAS was a known cytoplasmic DNA sensor that signals to upregulate interferon (IFN) expression to activate the anti-virus response [21, 22], we hypothesize that the micronuclei formed in the syncytial cytosol might be recognized by cGAS to activate IFN response. In line with this idea, immunostaining indicated a strong localization of cGAS on the micronuclei formed in syncytia induced by either spike transfection and SARS-CoV-2 infection (Fig. 3a). Interestingly, IRF3, the downstream effector critical for IFN expression, was also localized on the micronuclei along with nuclear translocation in SARS-CoV-2 induced syncytia (Fig. 3a, b). Quantitative analysis indicated that more than half of the micronuclei were positive in cGAS and IRF3 (Fig. 3c). Consistent with the typical subcellular localization pattern of cGAS and IRF3, the expression of IFN (IFNB1) and its downstream target genes (IFIT2, CCL5, CXCL10) were all significantly upregulated in cells forming syncytia upon spike expression as compared with control cells (Fig. 3d) in agreement with the activation of cGAS-STING signaling, the upregulated expression of IFN and its target genes took place concomitantly with increased expression and phosphorylation of cGAS, STING and IRF3 proteins as detected by Western blot (Fig. 3e, f).

## Discussion

**Fig. 4:**
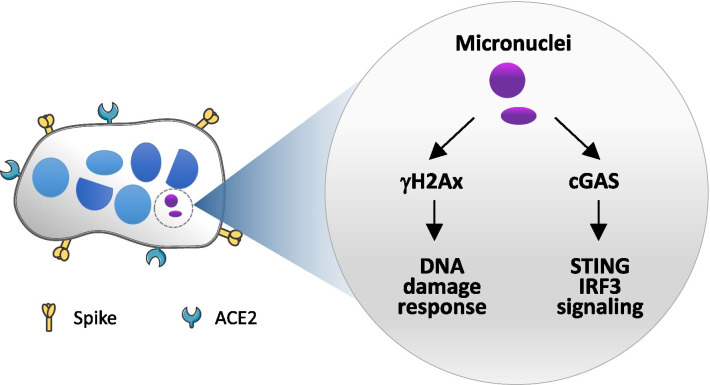
Schematic illustration of activation of cGAS and DDR signaling initiated from micronuclei

Together, our data fit well with a model where SARS-CoV-2 infection induced cell–cell fusion to form multinucleate syncytia in a way dependent on spike-ACE2 interaction. The formation of syncytia incurred the production of micronuclei that contain DNA damages, which allow micronuclei to recruit γH2Ax and cGAS, respectively, eventually leading to the activation of DNA damage response and cGAS-STING-IFN signaling (Fig. 4). Thus, our results provide a plausible explanation for tissue damages and excessive inflammation manifested in severe patients with COVID-19 at late stage. On the one hand, constant activation of both DNA damage response and cGAS-STING signaling promoted the death of syncytia, which contributes to tissue damage. On the other hand, activation of cGAS pathway upregulated the expression of IFN to promote local and systemic inflammation, which was further enhanced by the lysis of syncytia. Altogether, syncytium formation may serve as a unit to promote the pathogenesis of late stage COVID-19, and therefore a potential target for the therapy of severe COVID-19.

Interestingly, IFN expression was known as an antiviral response by inducing expression of a set of interferon-stimulated genes (ISGs) that endow antiviral activities to host cells [23]. Therefore, the syncytia-mediated activation of IFN response via cGAS-STING signaling would be beneficial for blocking SARS-CoV-2 infection at early stage. This is particularly important when the inherent anti-SARS-CoV-2 mechanism via RIG-I-MAVS-TBK1 signaling pathway was evaded by SARS-CoV-2 proteins during early infection [24]. For example, the structural M protein could bind MAVS to block its aggregation and promote TBK1 degradation [25, 26]; the non-structural protein NSP6 and NSP13 could bind and inhibit TBK1 activity [27]; ORF16 and NSP1 proteins could either block the nuclear translocation of IRF3, the downstream signal mediator of TBK, by binding to importin karyopherin alpha 2 [28], or shutdown mRNA translation of IFNs and ISGs by binding to 40S ribosomal subunit [29]. Consistent with this idea, activation of IFN response by STING agonist was reported to be effective in suppressing SARS-CoV-2 infection [30–32].

Our finding also fit well with the reports that patients with COVID-19 usually exhibited delayed type I IFN response, that is, IFN production was inhibited at early stage of SARS-CoV-2 infection, but substantially exaggerated at the late stage [33]. While the mechanism underlying the early inhibition were revealed as discussed above, relatively much less was known about the mechanisms underlying the activation of IFN response at late stage. Recently, Zhao et al. reported that the structural N protein may play a dual-role in regulating IFN signaling based on its expression level, i.e., the low-dose N protein was suppressive, while the high-dose was promotive, for the IFN response. This worked out by dually regulating the phosphorylation and nuclear translocation of IRF3, partially explaining how IFN signaling was activated [34]. As an alternative, our data provided an additional way for SARS-CoV-2 RNA virus to activate IFN response unexpectedly via cGAS-STING signaling pathway, which was secondary to the formation of multinucleate syncytia between cells expressing spike and ACE2. Since syncytium formation is a common phenomenon taking place during most viral infection, targeting syncytium formation and cGAS-STING signaling holds the promise to treat a variety of virological diseases where syncytia were induced.

## Materials and methods

### Cell culture

Hela, Hela-ACE2 cells were maintained in DMEM (MACGENE Tech Ltd., Beijing, China) supplemented with 10% fetal bovine serum (FBS) (ExCell Bio, Shanghai, China) plus a final concentration of 1% Penicillin–Streptomycin (MACGENE Tech Ltd., Beijing, China) or without antibiotics when transfection. Cells were incubated at 37 °C, 5% CO_2_.

### Constructs

The codon-optimized SARS-CoV-2 S cDNA was synthesized at Genscript Biotech Corporation (Nanjing, China). The wild type S gene of SARS-CoV-2 was cloned into pSecTag2-Hygro-A vector through seamless homologous recombination. Human ACE2 genes were cloned into the self-inactivating retroviral vector plasmids, pQCXIP-Puro to generate pQCXIP-ACE2-Puro. Retroviral helper plasmids VSV-G, Gap-pol-Rev were from Addgene. All constructs were verified by DNA sequencing with detail information as below:

**Table:** 

Plasmid name	Construct method	Vector backbone	Cutting site	DNA	Primer Sequence (5′ → 3′)
pSecTag2-COV2-S	Homologous recombination	pSecTag2 Hygro A	XhoI	COV2-S	AGCTTGGTACCGAGCTCgCAGTGCGTCAATCTGACAACTCG
			BamHI		TTCGGGCCCTCCTCGAGCGGTGTAATGCAGCTTCACGC
pQCXIP-ACE2	Homologous recombination	pQCXIP-VCL-Full length	SbfI	ACE2	CATTGGAACGGACCTGCAgccaccATGTCAAGCTCTTCC
			Not		attatgatctagagtcgCtcaCTTGTCATCGTCATCCTTGTAGTCg

### Generation of ACE2-expressing cells

For stable ACE2-expressing Hela cells, 293FT cells were co-transfected with the retroviral vector and retroviral helper plasmids to make retroviral particles that were subsequently used to infect Hela cells. Cells stably expressing ACE2 (Hela-ACE2) were selected in the presence of 8 μg/mL puromycin. The expression of ACE2 was confirmed by Western blot.

### Syncytium formation assay

Syncytium formation assay was performed as described previously [10] with slight modification. About 4.0 × 10^5^ Hela-ACE2 cells per well were seeding in 6-well plate precoated with type I collagen (354236, BD Bioscience). After 16 h culture, cells were then transfected with spike constructs by Lipofectamine LTX and PLUS™ reagent (2250382, Thermal Fisher Scientific, US) to induce syncytia.

The 2019-CoV-2 (GenBank ID: MT627325), a clinical isolate of SARS-CoV-2 virus, was propagated in Vero E6 cells, and viral titer was determined by 50% tissue culture infective dose (TCID50) using immunofluorescence assay. Hela-ACE2 cells (4 × 10^5^ cells/well) in 6-well Cell culture plate were first infected with SARS-CoV-2 (MOI of 0.1) for 24 h, and then cultured in normal medium overnight to form syncytia. All the SARS-CoV-2 infection experiments were performed in a biosafety level-3 (BLS-3) laboratory in the Department of Microbiology at the 2nd Military Medical University. Images of 4 random fields (10 × objective lens) were taken on Hoechst-stained cells 24 h post transfection by Nikon microscope. Nucleus counting was performed by NIS elements AR software (Nikon, Japan). The fusion index (FI) was calculated as “% of nuclei in fused cells”.

### Western blotting

Cells were lysed on ice with cold RIPA buffer containing phosphatase-protease and protease inhibitors (CWBiotech, Beijing) for 20 min followed by ultrasonic disruption (power 40%, work 6 s, pause 9 s, 4 times in total). After being centrifuged at 12,000 rpm for 10 min, the supernatant was collected for SDS-PAGE electrophoresis followed by transferring onto the PVDF membrane (0.2 μm, Millipore). The PVDF membrane, blocked with 5% skimmed milk (BD, USA) or 5% bovine serum albumin (BSA) (Sigma, USA) for 1 h at room temperature, was then blotted with primary antibodies in 5% BSA for 12 h at 4 °C or 4 h at room temperature, followed by one-hour secondary antibodies at room temperature.

The primary antibodies used: ACE2 (Proteintech, 1:3000, 66699-1-Ig), SARS-CoV2 (COVID-19) spike (GeneTex, 1:2000, GTX632604), Anti-STING antibody (Abcam, 1:1000, ab181125), Phospho-STING (Ser366) (E9A9K) (CST, 1:1000, 50907T), cGAS (CST, 1: 1000, 79978S), phospho-CGAS-Y215 (Abclonal, 1:1000, AP0946), IRF-3 (CST, 1:1000, 11904T), Phospho-IRF-3 (Ser386) (CST, 1:1000, 37829T), p53(DO-1) (Santa Cruz, 1:1000, Sc-126), β-Actin (Proteintech, 1:5000, 60008-1-lg), Phospho-Histone H2AX-S139 (Abclonal, 1:1000, AP0099). The secondary antibodies used: anti-rabbit IgG HRP (CST, 1:3000, #7074), anti-mouse IgG HRP (CST, 1:3000, #7076).

### Immunofluorescence and quantification

Cells were grown on glass coverslips and fixed in 4% paraformaldehyde in PBS for 10 min at room temperature, followed by 5-min washes for three times with PBS, and permeabilized with 0.2% Triton X-100 in PBS for 5 min. After three-time washing, cells were blocked in 5% BSA in PBS for 1 h at room temperature. Cells were then incubated with primary antibodies diluted in 5% BSA in PBS supplemented with 0.1% Tween 20 (PBST) overnight at 4 °C or 1 h at room temperature. Then, cells were washed four times with PBS, each for 10 min, followed by incubation with Alexa Fluor-conjugated secondary antibody (Life Technologies, USA), in 5% BSA/PBST for 1 h at room temperature.

The following primary antibodies were used for immunofluorescence: Phospho-Histone H2AX-S139 (Abclonal, 1:100, AP0099), IRF-3 (CST, 1:200, 11904T), cGAS (CST, 1: 200, 79978S). Secondary antibodies, anti-rabbit-Alexa488 (2256692), and anti-rabbit-Alexa647 (A11036) (Invitrogen), were used at 1:500 dilution. All slides were stained with DAPI (ZSGB-BIO, ZLI-9557) and Alexa Fluor®568 Phalloidin (1:200, Life technologies, A12379) to indicate nuclei and actin, respectively. Images were captured and processed by Ultraview Vox confocal system (Perkin Elmer) or Widefield Fluorescence system (Nikon, Japan) on Nikon Ti-E microscope. Blind scoring was performed.

### Reverse transcription-quantitative PCR (RT-qPCR)

Total RNA was extracted with RNAiso Plus (TaKaRa, Japan) according to the manufacturer’s manual. Reverse transcription (RT) was done using TransScript One-Step gDNA Removal and cDNA Synthesis SuperMix (TransGen Biotech, China), and then quantitative PCR (qPCR) was performed using SYBR Green Realtime PCR Master Mix (TOYOBO, Japan) on an qTOWER^3^G machine (Analytik Jena AG, Germany). The expression of target genes was normalized to the housekeeping gene GAPDH. The following primers (5′–3′) were used for RT-qPCR:

- IFIT2: AAGCACCTCAAAGGGCAAAAC, TCGGCCCATGTGATAGTAGAC;
- IFNβ1: GCTTGGATTCCTACAAAGAAGCA, ATAGATGGTCAATGCGGCGTC;
- CCL5: CCAGCAGTCGTCTTTGTCAC, CTCTGGGTTGGCACACACTT;
- CXCL10: TAAGTGGCATTCAAGGAGTA, TGGATTCAGACATCTCTTCTC;
- NOXA: GCTGGAAGTCGAGTGTGCTA, CCTGAGCAGAAGAGTTTGGA;
- GADD45A: AGAAGACCGAAAGCGACCC, GTTGATGTCGTTCTCGCAGC;
- SLC7A11: GCTGGGCTGATTTATCTTCG, GAAAGCTGGGATGAACAGT;
- PAI1: CCGCCGCCTCTTCCA, GCCATCATGGGCACAGAGA;
- MYC: GGCTCCTGGCAAAAGGTCA, CTGCGTAGTTGTGCTGATGT;
- GAPDH: GGAGCGAGATCCCTCCAAAAT, GGCTGTTGTCATACTTCTCATGG;

### Statistics

All data were plotted as averages with variance as standard error of the mean (SEM) unless stated otherwise. Statistical analysis was performed by Prism (Graphpad Software Inc.). For all quantitative measurements, normal distribution was assumed, t-tests were performed with unpaired and two-sided unless otherwise stated. At least three independent replicated were analyzed.

## Data Availability

Not applicable.
